# A Highly Contiguous and Annotated Genome Assembly of the Lesser Prairie-Chicken (*Tympanuchus pallidicinctus*)

**DOI:** 10.1093/gbe/evad043

**Published:** 2023-03-17

**Authors:** Andrew N Black, Kristin J Bondo, Andrew Mularo, Alvaro Hernandez, Yachi Yu, Carleigh M Stein, Andy Gregory, Kent A Fricke, Jeff Prendergast, Dan Sullins, David Haukos, Michael Whitson, Blake Grisham, Zach Lowe, J Andrew DeWoody

**Affiliations:** Department of Forestry and Natural Resources, Purdue University, West Lafayette, Indiana; Department of Natural Resources and Management, Texas Tech University, Lubbock, Texas; Department of Biological Sciences, Purdue University, West Lafayette, Indiana; Roy J. Carver Biotechnology Center, University of Illinois at Urbana-Champaign, Illinois; Roy J. Carver Biotechnology Center, University of Illinois at Urbana-Champaign, Illinois; Department of Biological Sciences, University of North Texas, Denton, Texas; Department of Biological Sciences, University of North Texas, Denton, Texas; Kansas Department of Wildlife and Parks, Emporia, Kansas; Kansas Department of Wildlife and Parks, Hays, Kansas; Horticulture and Natural Resources, Kansas State University, Manhattan, Kansas; U.S. Geological Survey, Kansas Cooperative Fish and Wildlife Research Unit, Kansas State University, Manhattan, Kansas; Department of Natural Resources and Management, Texas Tech University, Lubbock, Texas; Department of Natural Resources and Management, Texas Tech University, Lubbock, Texas; Western Association of Fish and Wildlife Agencies, Boise, Idaho; Department of Forestry and Natural Resources, Purdue University, West Lafayette, Indiana; Department of Biological Sciences, Purdue University, West Lafayette, Indiana

**Keywords:** de novo assembly, haploid, Pacific Biosciences, TELL-seq, conservation, prairie grouse

## Abstract

The Lesser Prairie-Chicken (*Tympanuchus pallidicinctus*; LEPC) is an iconic North American prairie grouse, renowned for ornate and spectacular breeding season displays. Unfortunately, the species has disappeared across much of its historical range, with corresponding precipitous declines in contemporary population abundance, largely due to climatic and anthropogenic factors. These declines led to a 2022 US Fish and Wildlife decision to identify and list two distinct population segments (DPSs; i.e., northern and southern DPSs) as threatened or endangered under the 1973 Endangered Species Act. Herein, we describe an annotated reference genome that was generated from a LEPC sample collected from the southern DPS. We chose a representative from the southern DPS because of the potential for introgression in the northern DPS, where some populations hybridize with the Greater Prairie-Chicken (*Tympanuchus cupido*). This new LEPC reference assembly consists of 206 scaffolds, an N50 of 45 Mb, and 15,563 predicted protein-coding genes. We demonstrate the utility of this new genome assembly by estimating genome-wide heterozygosity in a representative LEPC and in related species. Heterozygosity in a LEPC sample was 0.0024, near the middle of the range (0.0003–0.0050) of related species. Overall, this new assembly provides a valuable resource that will enhance evolutionary and conservation genetic research in prairie grouse.

**Significance**

On November 25, 2022, the US Fish and Wildlife Service listed the Lesser Prairie-Chicken’s (LEPC) northern distinct population segment as “threatened” and the southern DPS as “endangered” under the 1973 Endangered Species Act (United Sates Office of the Federal Registry 2022). The annotated genome assembly presented herein will facilitate future assessments of population structure, genomic diversity, sexual selection, local adaptation, and evolutionary history in the LEPC, other prairie grouses, and their hybrids.

## Introduction

The Lesser Prairie-Chicken (*Tympanuchus pallidicinctus*; LEPC) is a North American prairie grouse renowned for its extravagant breeding behavior on lekking grounds (Hagen et al. 2020). However, the distribution of the LEPC-occupied range has contracted since European settlement, with accelerated population declines during the late 1980s (Hagen et al. 2013; Haukos and Boal 2016). The decline in LEPC population abundance and occupied range has been attributed to habitat loss and degradation resulting from the conversion of native prairie to cropland, anthropogenic development, climate change, and introduction and spread of invasive plant species (Hagen and Giesen 2005; Boal and Haukos 2016; Rodgers 2016; Van Pelt 2016). Currently, LEPC populations occupy landscapes across five states: Colorado, New Mexico, Oklahoma, Kansas, and Texas. Recently (November 25, 2022), the US Fish and Wildlife Service separated the LEPC range into two distinct population segments (DPSs), listing the southern DPS (eastern New Mexico and southwest Texas Panhandle) as endangered and the northern DPS (southeast Colorado, Kansas, western Oklahoma, and northeast Texas Panhandle) as threatened (for a map of LEPC distribution, see United States Office of the Federal Registry 2022). We report the first de novo genome assembly and annotation of a LEPC sample collected from the southern DPS. Currently, the only other *Tympanuchus* species with a reference genome on the National Center for Biotechnology Information (NCBI) is the Greater Prairie-Chicken (*Tympanuchus cupido*; GRPC), which has an N50 of 12 Mb among 2,186 scaffolds and is currently unannotated (GCA_001870855.1).

## Results and Discussion

### De Novo Genome Assembly and Annotation

**Fig. 1.**
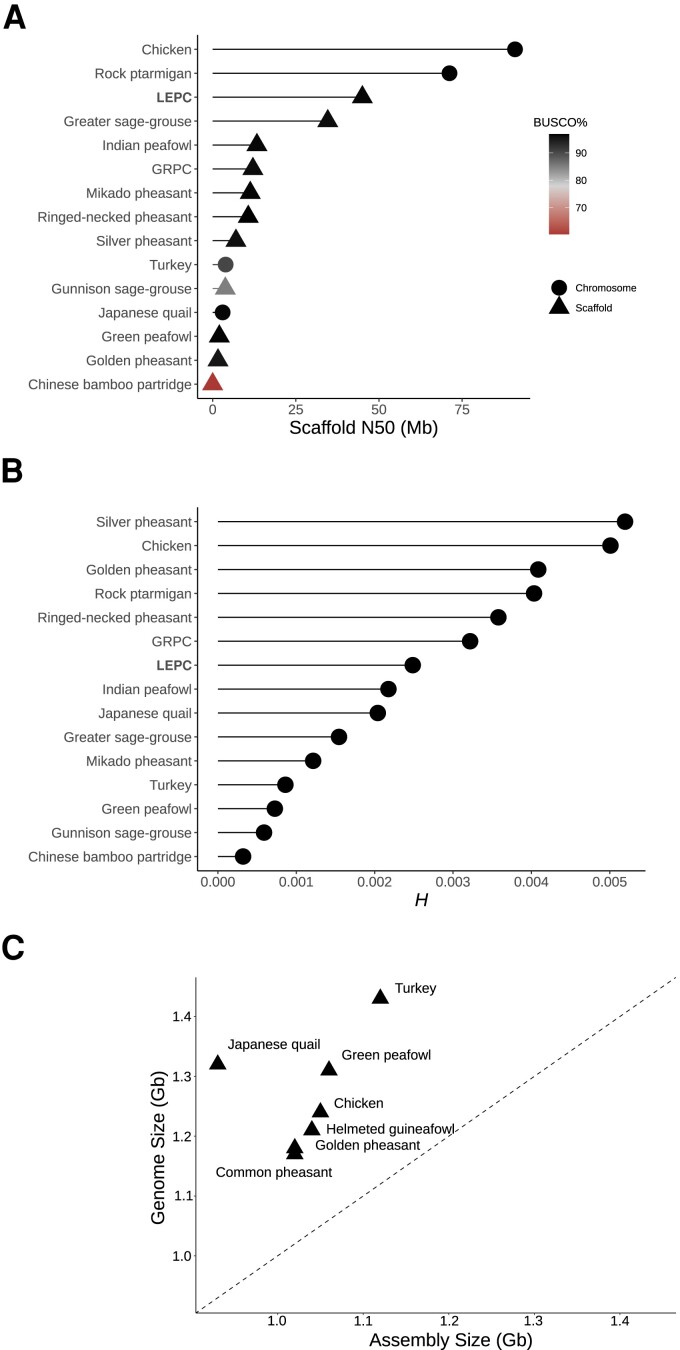
Genome statistics for the new LEPC genome (bold) and related avian species (Phasianidae) with publicly available reference genome assemblies and associated Illumina sequence data. (*A*) Genome contiguity (N50) for the most current accession of each species, with assembly build (chromosome or scaffold level) and Avian BUSCO % signified in symbol shape and color, respectively. (*B*) Genome-wide heterozygosity (*H*) derived from the alignment of Illumina shotgun data (*N* = 15 species). The LEPC and GRPC samples were sequenced in the current study for estimates of *H*. (*C*) Relationship between estimated genome size and assembly size for species in the Family Phasianidae where both haploid nuclear DNA content and genome assemblies are available from the Animal Genome Size Database and NCBI, respectfully, as of 27 February 2023. Genome Size (Gb) was estimated by multiplying the *C*-value of each species by 0.978 (Gregory 2023). In cases where a particular species had more than one *C*-value, a mean value was used. Assembly size (Gb) was obtained from the total genome length (including gaps) of the most current assembly for each species. The dashed black line represents a (theoretical) perfect correlation between *C*-value length and genome assembly size. In all cases, the assembly size was substantially smaller than the genome size derived from haploid nuclear DNA content.

Two SMRTcells of PacBio circular consensus reads (CCRs) yielded 65.8 Gb among 4 M reads with a mean read length of 14 kb. Analysis of 60-mers predicted a 0.987 Gb haploid genome length, with a heterozygosity of 0.0037 (i.e., 0.37% of the 987 Mb were heterozygous). Following de novo assembly, 64 contigs were identified as haplotigs and removed from the assembly. This resulted in 273 contigs with a N50 of 19 Mb and genome length of 0.995 Gb. A NovaSeq 6000 run yielded 900 M Tell-seq reads, which were then used to scaffold the contigs. This resulted in 215 scaffolds, with a N50 of 45 Mb and haploid genome length of 0.994 Gb. All scaffolds were assigned to Phasianidae except for nine, which were taxonomically unclassified. These nine scaffolds were highly repetitive, totaled 641 kb in length and had a skewed GC content (0.49), so were removed from the assembly. This resulted in a final curated genome assembly size of 0.945 Gb (close to the *k*-mer-based prediction length of 0.987 Gb) among 206 scaffolds with a N50 of 45.3 Mb (table 1). This is approximately 74% of the genome size of related species (family Phasianidae) as estimated based on cellular DNA content, where the mean genome size is 1.28 Gb (Gregory 2023; fig. 1*C* and table 1). The disparity between assembly size and genome size (as based on cellular DNA content) difference in size is most likely due to the exclusion of highly repetitive regions of the genome based on assembly algorithms, an aspect of genomics in nonmodel species that is rarely recognized.

**Table 1:** LEPC Genome Assembly and Annotation Summary

Assembly	Draft genome size (Mb)	0.994
	Depth of coverage (×)	*93.5*
	Number of scaffolds	*206*
	Longest scaffold (Mb)	*108*
	N50 (Mb)	*45.4*
	GC (%)	*41*
BUSCO	Complete BUSCOs (%)	*96.4*
	Complete and single-copy BUSCOs (%)	*96.1*
	Complete and duplicated BUSCOs (%)	*0.3*
	Fragmented BUSCOs (%)	*0.06*
	Missing BUSCOs (%)	*3*
Annotation	Total number of genes	*20,698*
	Length of genes (mean [bp])	*32,096*
	Number of protein-coding genes	*15,563*
	Number of transcripts per gene (mean)	*2.54*
	Number of exons per gene (mean)	*12.87*

Note.—Benchmarking Universal Single-Copy Orthologs (BUSCO) is used to assess the completeness of nuclear sequences.

The diploid number varies in Phasianidae (2*n* = 50–82; Degrandi et al. 2020), but many of the largest scaffolds likely represent full chromosomes. For example, the longest LEPC scaffold is 108 Mb, similar in size to the Rock ptarmigan (*Lagopus muta*) chromosome 2 (110 Mb). Short LEPC scaffolds may represent partial chromosomes that were bioinformatically split due to repetitive elements in centromeric regions. When comparing genome contiguity of our new LEPC assembly to other Phasianidae with associated sequence reads archive (SRA) data in NCBI, the LEPC genome ranks third highest, behind Rock ptarmigan and Chicken (*Gallus gallus*) chromosome-level assemblies (fig. 1*A*). Benchmarking Universal Single-Copy Orthologs (BUSCO) analysis of nucleotide sequence data identified 96.4% of Phasianidae orthologs in the LEPC genome assembly, which is in the top ranks of genome completeness observed in Phasianidae genomes (range = 60.4–96.7%; fig. 1*A*).

The NCBI Eukaryotic Genome Annotation Pipeline identified 20,698 total genes with a mean length of 32,096 bp (table 1). Of these, 15,563 protein-coding genes contained 98.7% of the Avian orthologs, which were classified as complete (98.5% single copy, 0.2% duplicated), 0.4% as fragmented, and 1% as missing. Furthermore, 40,823 fully supported mRNAs and 9,527 fully supported noncoding mRNAs were annotated in the LEPC scaffold level assembly.

### Genetic Diversity

Illumina shotgun sequencing of one LEPC sample and one GRPC sample sequenced as part of a related study yielded 11.6 T nucleotides among 38.8 M paired-end reads. The LEPC reads had an alignment rate of 97.40% to the new LEPC genome assembly, whereas the GRPC reads had an alignment rate of 97.38% to the existing GRPC assembly. The LEPC/GRPC had an average coverage of 4.11/4.17 and an 1× breadth of 90.80%/90.32%, respectively. Of the 13 SRA paired-end fastq files downloaded from related species in the family Phasianidae, species had an average (min–max) mapping rate of 96% (89.35–100%), coverage of 22.8× (4.5–71.1×), and breadth of 96.1% (89–99.1%). Due to the high variation in average coverage rates among the sampled Phasianidae, reads were subsampled for a target depth of coverage of 7× for each species; this resulted in mean depth of coverage of 6.76 (4.50–8.41). By determining the proportion of heterozygous sites for each sampled species, genome-wide heterozygosity (*H*) was then estimated for each species using a single representative sample. Estimates ranged from *H* = 0.000325 (Chinese bamboo partridge; *Bambusicola thoracicus*) to *H* = 0.005 (Silver pheasant; *Lophura nycthemera*). Comparing *H* pre- and postsubsampling for read depth of coverage showed *H* was highly positively correlated (*r* = 0.995). That is, subsampling for unequal depth of coverage had little to no effect on heterozygosity estimates in the sampled Phasianidae. Both the newly sequenced LEPC (*H* = 0.0024) and GRPC (*H* = 0.0032) sample had similar heterozygosities that were intermediate compared with related species (fig. 1*B*).

The LEPC we chose as a representative individual for the reference genome assembly was sampled from the southern DPS in New Mexico, which has lower levels of microsatellite heterozygosity than the northern DPS (Oyler-McCance et al. 2016). Interspecific hybridization between GRPC and LEPC occurs in areas of overlap in the northern DPS, where hybridization rates are estimated at ∼5% of matings at areas of sympatry in west central Kansas (Dahlgren et al. 2016). The range of the LEPC in the Southern DPS does not overlap with that of the GRPC, so there is little chance of recent hybrid background in the southern DPS. A second LEPC sample had a heterozygosity estimate (*H* = 0.0024) similar to that estimated using a *k*-mer-based approach (*H* = 0.0037). The GRPC sampled in Kansas showed similar, but slightly higher, heterozygosity (*H* = 0.0032). Future, and ongoing, population-based heterozygosity estimates will determine if both species have similar levels of diversity and the distribution of that diversity in space and time.

## Materials and Methods

### Sample Collection and Sequencing

A single male LEPC (sample G-5309 internally cataloged as F22 at Purdue University) collected on April 30, 2019 from Chaves County, New Mexico was selected as a representative individual for the reference genome assembly due to sample quality and geographic location. Genomic DNA (gDNA) was extracted from blood in lysis buffer using a MagAttract HMW DNA kit (Qiagen). The gDNA was then sheared to an average fragment length of 13 kb using a Megaruptor 3 (Diagenode). Sheared fragments were converted to libraries using the SMRTBell Express Template prep kit (3.0) and sequenced across two Sequel II SMRTcells. TELL-seq libraries were prepared using the whole-genome sequencing library prep kit (Universal Sequencing), pooled, quantitated by quantitative polymerase chain reaction (PCR), and sequenced on one SP lane for 151 cycles on a NovaSeq 6000.

To generate genomic diversity estimates, a second male LEPC was collected from Chaves County New Mexico on May 11, 2019 (sample G5397 internally labeled F21) and a single male GRPC collected from Wilcox County Kansas on April 19, 2020 (sample TK216221 internally labeled as F334). gDNA was extracted using the MagAttract HMW DNA kit (Qiagen) and assessed using Qubit dsDNA assays and Femto Pulse (Agilent, CA, USA). Shotgun libraries were prepared using the Illumina DNA Prep kit (Illumina) and sequenced with an S4 flowcell on an Illumina® NovaSeq™ 6000. All benchworks were conducted at the Roy J. Carver Biotechnology Center, University of Illinois at Urbana-Champaign.

### De Novo Genome Assembly and Annotation

PacBio CCRs from both SMRTcells were generated using Smrtlink (v.11.0) with the following parameters: ccs –min-passes 3 –min-rq 0.99. TELL-seq files were generated and demultiplexed with Bcl2fastq (v2.20; Illumina) and trimmed using the default parameters in Tellread (v.1.03; Universal Sequencing). To inform genome assembly and provide context for diversity metrics, *k*-mer counting was conducted with Jellyfish (v.2.30; Marçais and Kingsford 2011) using canonical (-C) 60-mers. A histogram of *k*-mer counts was then used to estimate genome assembly size and heterozygosity with GenomeScope (Vurture et al. 2017).

Circular consensus reads were assembled using Nextdenovo (https://github.com/Nextomics/NextDenovo), specifying the following parameters: genome_size = 1 g, read_type = hifi, min_read_len = 1k, -k17, -w17, -a 1. Following de novo assembly of CCR, haplotigs were identified and removed using Purge_dups (Guan et al. 2020). Tell-seq reads were then used to scaffold contigs from each assembly using the *k*-mer approach (*k* = 60) implemented in Arks (Coombe et al. 2018). Following assembly, reads were screened for nontarget sequence reads with Blobtools (Laetsch and Blaxter 2017). Following curation of the genome assembly, repeat masking and gene annotation were completed using the NCBI Eukaryotic Annotation Pipeline and assembly completeness was evaluated with BUSCO using 8,338 orthologs in the Avian database (v. 5.4.1; Mani et al. 2021).

### Genetic Diversity

Genomic diversity point estimates were generated for the LEPC, GRPC, and 13 other species from the family Phasianidae (i.e., those with reference assemblies available in the NCBI as of October 1, 2022). Reference genomes and associated SRA data were downloaded for each assembly (supplementary table S1, Supplementary Material online). For species where SRA files were not publicly available, Illumina whole-genome shotgun data were selected from a different biosample. For each species, paired-end (2 × 151 bp) Illumina reads were aligned to unambiguous nonrepetitive regions in sequences ≥100 kb for each genome assembly using the Bwa (v.0.7.17; Li 2013) mem algorithm. Ambiguous regions were determined by calculating mappability (<1) for each reference genome using Genmap (v.1.3.0; Pockrandt et al. 2020), which was run using 100-mers and allowing up to two mismatches. Repetitive regions were determined by running Repeatmasker (v.4.07; Smit et al. 2015) using the Phasianidae repeat library. Following alignment, Samtools (v.1.8; Li et al. 2009) bitflags (italicized) were used to remove unmapped (*4*), secondary (*256*), QC failed (*512*), duplicate (*1,024*), and supplementary (*2,048*) alignments. Alignments were sorted based on read coordinate, and PCR duplicates were removed using PicardTools (Broad Institute 2019). The mapped reads were realigned around insertions/deletions using Gatk3 (v.3.6.0; McKenna et al. 2010) and Samtools was used to subsample high-coverage samples to a target depth of 7×. Angsd (v.095; Korneliussen et al. 2014) was used to calculate genotype likelihoods from the BAM files using the Samtools model (-GL 1) after removing low-quality alignments/bases (-minMapQ 30, -minQ 30). Individual heterozygosity (i.e., proportion of heterozygotes; *H*) was determined from the site-frequency spectrum and plotted for all samples using the R (R Core Team 2022) package *ggplot2* (Wickham 2016).

## Supplementary Material

evad043_Supplementary_Data

## Data Availability

Unprocessed sequence data have been archived to the NCBI Sequence Read Archive under Bioproject PRJNA910496. The assembled Refseq genome can be found under accession GCF_026119805.1 along with NCBI *T. pallidicinctus* Annotation Release 100.
