# Supplementary material for: A Highly Contiguous and Annotated Genome Assembly of the Lesser Prairie-Chicken (Tympanuchus pallidicinctus)
Source: Genome Biol Evol. 2023 Mar 17;15(4):evad043. doi: 10.1093/gbe/evad043 (PMC10118296; doi:10.1093/gbe/evad043)
Supplement: evad043_Supplementary_Data [file evad043_supplementary_data.docx]

| **Species Name** | **Name** | **Accession** | **Assembly Length (Gb)** | **SRA** |
| --- | --- | --- | --- | --- |
| *Gallus gallus*   (chicken) | bGalGal1.mat.broiler.GRCg7b | GCF_016699485.2 | 1.05 | SRR1291159 |
| *Lagopus muta*  (Rock ptarmigan) | bLagMut1 primary | GCF_023343835.1 | 1.03 | SRR8393387 |
| ***Tympanuchus pallidicinctus***  **(LEPC)** | **pur_lepc_1.0** | **GCF_026119805.1** | **0.99** | **NA** |
| *Centrocercus urophasianus*  (Greater sage-grouse) | USGS_Curo_1.0 | GCA_019232065.1 | 1.01 | SRR8863287 |
| *Tympanuchus*  *cupido pinnatus*  (GRPC) | T_cupido_pinnatus_GPC_3440_v1 | GCA_001870855.1 | 0.98 | NA |
| *Syrmaticus mikado*  (Mikado pheasant) | NTU_Smik_1.2 | GCA_003435085.1 | 1.06 | SRR5666076 |
| *Lophura nycthemera*  (Silver pheasant) | ASM2129221v1 | GCA_021292215.1 | 1.01 | SRR12459249 |
| *Meleagris gallopavo*  (turkey) | Turkey_5.1 | GCF_000146605.3 | 1.12 | SRR453269 |
| *Centrocercus minimus*  (Gunnison sage-grouse) | Cmin_1.0 | GCA_005890655.1 | 1.0 | SRR8863311 |
| *Coturnix japonica*  (Japanese quail) | Coturnix japonica 2.1 | GCF_001577835.2 | 0.93 | SRR2159508 |
| *Pavo muticus*  (Green peafowl) | ASM1664771v1 | GCA_016647715.1 | 1.06 | SRR12223802 |
| *Bambusicola thoracicus*  (Chinese bamboo-partridge) | Bthov1.0 | GCA_002909625.1 | 1.03 | SRR6466369 |
| *Chrysolophus pictus*  (Golden pheasant) | Chrysolophus_pictus_GenomeV1.0 | GCA_003413605.1 | 1.02 | SRR7647150 |
| *Pavo cristatus*  (Indian peafowl) | PavCris_1.0 | GCA_021513735.1 | 1.05 | SRR4068854 |
| *Phasianus colchicus*  (Ring-necked pheasant) | ASM414374v1 | GCF_004143745.1 | 1.02 | SRR10159563 |

**Table S1:** Avian species (Phasianidae) analyzed to compare Lesser Prairie-Chicken (LEPC) genome (in bold) contiguity/completeness and genome-wide heterozygosity. All Phasianidae genomes with associated Sequence Read Archive (SRA) Illumina data were downloaded from NCBI on October 1, 2022. For each genome, the Genus species and common name are provided, as well as the name of the assembly / accession used, the haploid assembly length (Gb), and the SRA file downloaded for diversity estimates. The Greater Prairie-Chicken (GRPC) and LEPC Illumina reads were sequenced as a part of our ongoing study, so there are no SRA accession numbers.
